# Gammaherpesvirus BoHV-4 infects bovine respiratory epithelial cells mainly at the basolateral side

**DOI:** 10.1186/s13567-019-0629-z

**Published:** 2019-02-08

**Authors:** Bo Yang, Jiexiong Xie, Jolien Van Cleemput, Ruifang Wei, Geert Opsomer, Hans J. Nauwynck

**Affiliations:** 1 Department of Virology, Parasitology and Immunology, Faculty of Veterinary Medicine, Ghent University, Salisburylaan 133, 9820 Merelbeke, Belgium; 2 Department of Reproduction, Obstetrics and Herd Health, Faculty of Veterinary Medicine, Ghent University, Salisburylaan 133, 9820 Merelbeke, Belgium

## Abstract

Bovine herpesvirus 4 (BoHV-4) is a gammaherpesvirus that is widespread in cattle. However, only a few studies about the pathogenesis of BoHV-4 primary infection have been reported. In the present study, ex vivo models with bovine nasal and tracheal mucosa explants were used to study the cellular BoHV-4-host interactions. Infection was observed in nasal but not in tracheal epithelial cells. To find a possible correlation between the integrity and restricted infection of the respiratory epithelium, both nasal mucosal and tracheal explants were treated with EGTA, a drug that disrupts the intercellular junctions, before inoculation. The infection was analyzed based on the number of plaques, plaque latitude and number of infected single cells, as determined by immunofluorescence. BoHV-4 infection in nasal mucosal explants was enhanced upon opening the tight junctions with EGTA. Infection in tracheal explants was only found after treatment with EGTA. In addition, primary bovine respiratory epithelial cells (BREC) were isolated, grown at the air–liquid interface and infected either at the apical or basolateral side by BoHV-4. The results showed that BoHV-4 preferentially bound to and entered BREC at the basolateral surfaces of both nasal and tracheal epithelial cells. The percentage of BoHV-4 infection was significantly increased both from nasal and tracheal epithelial cells after treatment with EGTA, which indicates that the BoHV-4 receptor is mainly located at the basolateral surface of these cells. Thus, our findings demonstrate that integrity of the respiratory epithelium is crucial in the host’s innate defense against primary BoHV-4 infections.

**Electronic supplementary material:**

The online version of this article (10.1186/s13567-019-0629-z) contains supplementary material, which is available to authorized users.

## Introduction

Bovine herpesvirus 4 (BoHV-4) is a member of the family *Herpesviridae*, subfamily *Gammaherpesvirinae*, genus *Rhadinovirus* [1]. BoHV-4 was isolated for the first time from animals with respiratory and ocular signs in Europe in 1963 [2]. BoHV-4 is widespread in bovine and remains latent and asymptomatic in the vast majority of infected animals. Viral replication can be reactivated by corticosteroids or stress, both factors present at calving [3]. Although BoHV-4 has been demonstrated in many tissues, accumulated evidence suggests that cells of the monocyte/macrophage lineage are the main site of persistence in both natural and experimental hosts [4].

There are several innate mucosal barriers between gammaherpesviruses and their hosts, which include the mucus layer, the mucociliary escalator, antimicrobial peptides and firm intercellular connections [5]. The airway surface liquid (ASL), often referred to as mucus, is the first layer of defense against incoming pathogens through mucociliary clearance. Intercellular junctions (ICJ) of the respiratory epithelium are crucial in the host’s innate defense against primary infection with alphaherpesvirus equine herpesvirus type 1 (EHV-1) [6]. Therefore, we hypothesized that intercellular junctions (ICJ) may play a similar important role for gammaherpesviruses in protecting the respiratory mucosa from primary replication. ICJ are specialized regions of contact between the plasma membranes of adjacent cells and form the apical cell domain, separating the external environment from the basolateral cell domains, which contacts the underlying cells and systemic vasculature [7].

Virus binding and subsequent entry may occur selectively at either the apical or basolateral domains of polarized cells, due to the specific expression of receptors required for binding and internalization. Some viruses, such as simian virus, hepatitis A virus and West Nile virus preferentially infect polarized cells at the apical surfaces [8–10], while vesicular stomatitis virus (VSV), Semliki Forest virus and EHV-1 prefer basolateral surfaces [6, 11, 12]. In respiratory epithelial cells, polarity of infection and the importance of ICJ have not been studied for gammaherpesviruses. Previous studies with a continuous cell line do not really reflect the in vivo situation [13, 14]. Therefore, a respiratory mucosal explant model, which mimics the in vivo situation, was used to investigate the importance of ICJ for the respiratory infection of the gammaherpesvirus BoHV-4. In addition, primary bovine respiratory epithelial cells (BREC) were isolated and cultivated on transwells to illustrate the polarity of BoHV-4 binding and subsequent viral replication.

In a previous study, ex vivo models with bovine genital tract mucosa explants were set up to elucidate the mucosal dissemination and invasion of BoHV-4 [15]. BoHV-4 replicates in the epithelial cells of uterus, cervix and vagina in a plaquewise manner and does not cross the basement membrane. Instead, it hijacks individual CD172a^+^ monocytic cells to invade the underlying connective tissue. During this migration, the BoHV-4 replication is silenced, because fibrocytes do not become infected. When BoHV-4 become produced in connective tissue (e.g. upon reactivation), fibrocytes may become infected and may eventually lead to pathological processes [15]. In the present study, respiratory mucosal explants and BREC were used to describe the invasion mechanism of gammaherpesvirus BoHV-4.

## Materials and methods

### Virus strain

The BoHV-4 strain V.test was used in this study, which belongs to the European clade of BoHV-4 strains. It was originally isolated from an infertile bull’s testicle. The strain V.test had previously received an unknown number of passages. The virus was passaged three times in Madin Darby Bovine Kidney (MDBK) cells in our laboratory.

### Tissue collection and processing

The nasal and tracheal mucosae were collected from healthy cattle at the local slaughterhouse and were immediately placed in phosphate buffered saline (PBS), supplemented with 1000 U/mL penicillin (Continental Pharma, Puurs, Belgium), 1 mg/mL streptomycin (Certa, Braine l’Alleud, Belgium), 1 μg/mL gentamycin (Invitrogen, Paisley, UK) and 5 μg/mL fungizone (Bristol-Myers Squibb, New York, USA) on ice for transportation to the laboratory. Nasal septum mucosal explants and trachea mucosal explants were prepared as previously described [15]. Primary bovine nasal and tracheal epithelial cells were isolated and cultured as described by Van Cleemput et al. [6].

### Disruption of intercellular junctions

#### Respiratory mucosa explants

Nasal and tracheal mucosa explants were cultured 24 h in medium [15]. Afterwards, the apical surface of the epithelial cells was exposed for 1 h at 37 °C to 8 mM ethylene glycol tetra-acetic acid (EGTA) (VWR International, Leuven, Belgium) as described by Galen et al. [16]. PBS was used as a control. Finally, explants were washed 3 times to remove excess of EGTA before inoculation. To determine the viability of the cells in the explants after treatment with EGTA, an in situ Cell Death Detection Kit (Roche Diagnostics Corporation, Basel, Switzerland) was used based on terminal deoxynucleotidyl transferase dUTP nick end-labeling (TUNEL).

#### Respiratory epithelial cells

Nasal and tracheal epithelial cells were grown in transwells. The medium from the wells was changed every day until confluency. After that, the cells were treated with 8 mM EGTA in PBS for 30 min prior to inoculation; PBS was used as a control. The viability of the cells was assessed by ethidium monoazide bromide (EMA) staining. EGTA treatment did not cause a significant cell death.

### Virus inoculation

#### Respiratory mucosal explants

After 24 h of cultivation of explants at the air-liquid interface, nasal and tracheal explants were treated with EGTA to dissociate ICJ or PBS as described above. Explants were submerged in 0.5 mL of BoHV-4 containing medium (10^7^ TCID_50_/mL) and incubated for 1 h (37 °C, 5% CO_2_). Before explants were placed back on the gauze, they were washed three times with PBS. The inoculated tissues were collected at 0 h, 24 h, 48 h and 72 hours post-infection (hpi). All gathered explants were embedded in cryoprotection medium [Methocel^®^, Fluka (Sigma)] and then frozen at −70 °C.

#### Respiratory epithelial cells

After respiratory epithelial cells became confluent in a transwell cell culture system, the cells were treated with EGTA or mock treated with PBS. Afterwards, the cells were inoculated with 100 µL of BoHV-4 (MOI = 2) at either the apical or the inverted basolateral surface for 1 h (37 °C, 5% CO_2_). The cells were washed 3 times with DMEM/F12. Fresh respiratory epithelial cell culture medium was added to each well and cells were further incubated at the air–liquid interface. The cells were collected at 0 h, 24 h, 48 h and 72 hpi and fixed in methanol for 20 min at −20 °C and stored dry at −20 °C until staining. The supernatant was used for titration.

### Binding assay

The virus-binding studies were carried out to characterize the attachment of BoHV-4 to respiratory epithelial cells. After treated with EGTA or PBS, the cells were chilled on ice for 5 min and washed 3 times with cold PBS. Then, BREC were inoculated at an MOI of 10 with BoHV-4 at either the apical or inverted basolateral surfaces for 1 h at 4 °C. The cells were washed 3 times with cold PBS to remove unbound virus and then fixed in methanol for 10 min. A primary mouse monoclonal IgG2a antibody (Mab35) against the early-late glycoprotein complex gp6/gp10/gp17 of BoHV-4 (1:1000 in PBS) was added. Next, cells were washed and incubated in a secondary goat anti-mouse IgG2a Alexa fluor^®^ 488 (Invitrogen) (1:200). Nuclei were counterstained with Hoechst 33342 (10 µg/mL; Invitrogen) for 10 min at room temperature and the transwell was mounted with glycerol-DABCO. The total number of virus particles attached to the apical or basolateral surfaces was counted. The percentage of BREC with bound BoHV-4 particles was calculated based on the number of cells with viral particles bound on the plasma membrane of 300 randomly selected cells.

### Virus titration

BREC culture medium were collected at 0 h, 24 h, 48 h and 72 hpi for virus titration. MDBK cells were inoculated for 1 h (37 °C, 5% CO_2_) with serial 10-fold dilutions (10^0^ to 10^−7^ in quadruplicate) of culture medium of BoHV-4 and mock inoculated BREC. Afterwards, MDBK cells were observed daily for cytopathic effect (CPE) for   7–9 days. The virus titer was calculated using the method of Reed and Muench [17].

### Immunofluorescent staining and confocal microscopy

#### Respiratory mucosal explants

At least 100 cryosections (20 μm) were made for each condition of both BoHV-4 and mock inoculated respiratory mucosae. Cryosections were fixed in methanol (−20 °C, 100%) for 20 min and washed in PBS. Afterwards, they were incubated with a primary mouse monoclonal IgG2a antibody (Mab35) against BoHV-4 for 1 h at 37 °C, followed by an incubation with a secondary goat anti-mouse IgG2a Alexa fluor^®^ 488 (Invitrogen) (1:200).

#### Respiratory epithelial cells

In order to identify the BoHV-4 infected respiratory epithelial cells, double immunofluorescence stainings were performed using different cell markers. Cells were incubated with a primary mouse monoclonal IgG2a antibody (Mab35) against BoHV-4 for 1 h at 37 °C, followed by an incubation with a secondary goat anti-mouse IgG2a Alexa fluor^®^ 594 (Invitrogen) (1:200). Afterwards, a monoclonal mouse IgG1 anti-human cytokeratin (Dako, Glostrup, Denmark) (1:50 in PBS) was added, followed by a secondary goat anti-mouse IgG1 FITC^®^ (Abcam, Cambridge, UK) (1:200 in PBS) to stain epithelial cells. Finally, nuclei were counterstained with Hoechst 33342 (10 µg/mL; Invitrogen) for 10 min at room temperature and cells were mounted with glycerol-DABCO. Analysis was performed by a TCS SPE confocal system (Leica Microsystems GmbH, Wetzlar, Germany).

### Identification of single infected cells in the lamina propria

In order to identify the single BoHV-4 infected cells after inoculation, a double immunofluorescence staining was performed using different markers. Firstly, cryosections were incubated by a mixture of a primary mouse monoclonal IgG2a antibody (Mab35) against BoHV-4 mixed and a primary monoclonal mouse IgG1 antibody DH59B (VMRD Inc., Pullman) directed against CD172a cells of the monocyte lineage for 1 h at 37 °C, followed by an incubation with a mixture of a secondary goat anti-mouse IgG2a Alexa fluor^®^ 594 (Invitrogen) and a secondary goat anti-mouse IgG1 FITC (Abcam, Cambridge, UK). Afterwards, nuclei were counterstained with Hoechst 33342 (10 µg/mL; Invitrogen) for 10 min at room temperature and slides were mounted with glycerol-DABCO. Analysis was performed by a TCS SPE confocal system (Leica Microsystems GmbH, Wetzlar, Germany).

### Statistical analysis

Data were statistically processed by Graphpad Prism 5.0 (GraphPad Software, Inc., San Diego, CA, USA) for analysis of variance (ANOVA). The data are represented as means with standard deviation (SD) of three independent experiments. Results with *p* values of < 0.05 were considered significant.

## Results

### BoHV-4 infects bovine nasal but not tracheal mucosa explants

BoHV-4 dissemination was examined in the established bovine respiratory mucosa explant system. Nasal and tracheal mucosae from three different animals were included and inoculated with BoHV-4. The explants were collected at different time points (0 h, 24 h, 48 h and 72 hpi).

**Figure 1:**
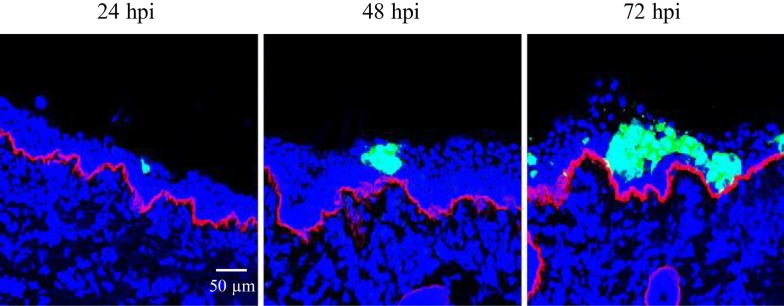
**Representative micrographs of BoHV-4 (green) replication at 24, 48** **h and 72** **hpi in bovine respiratory nasal mucosa explants.** The BM is visualized by mouse anti-collagen VII antibodies and goat anti-mouse Texas Red^®^ conjugate (red). Cell nuclei were counterstained with Hoechst (blue).

For the nasal explants, BoHV-4 positive cells were found at all collected time points post-inoculation. At 24 hpi, single positive cells or cluster of a few cells were observed in the epithelium and lamina propria. BoHV-4 positive plaques were detected in the epithelium at 48 and 72 hpi. The plaque did not cross the basement membrane (Figure 1). No infection was observed in the tracheal explants. These results show that BoHV-4 infects nasal but not tracheal mucosa explants.

### BoHV-4 cell tropism after inoculation in the lamina propria

**Figure 2:**
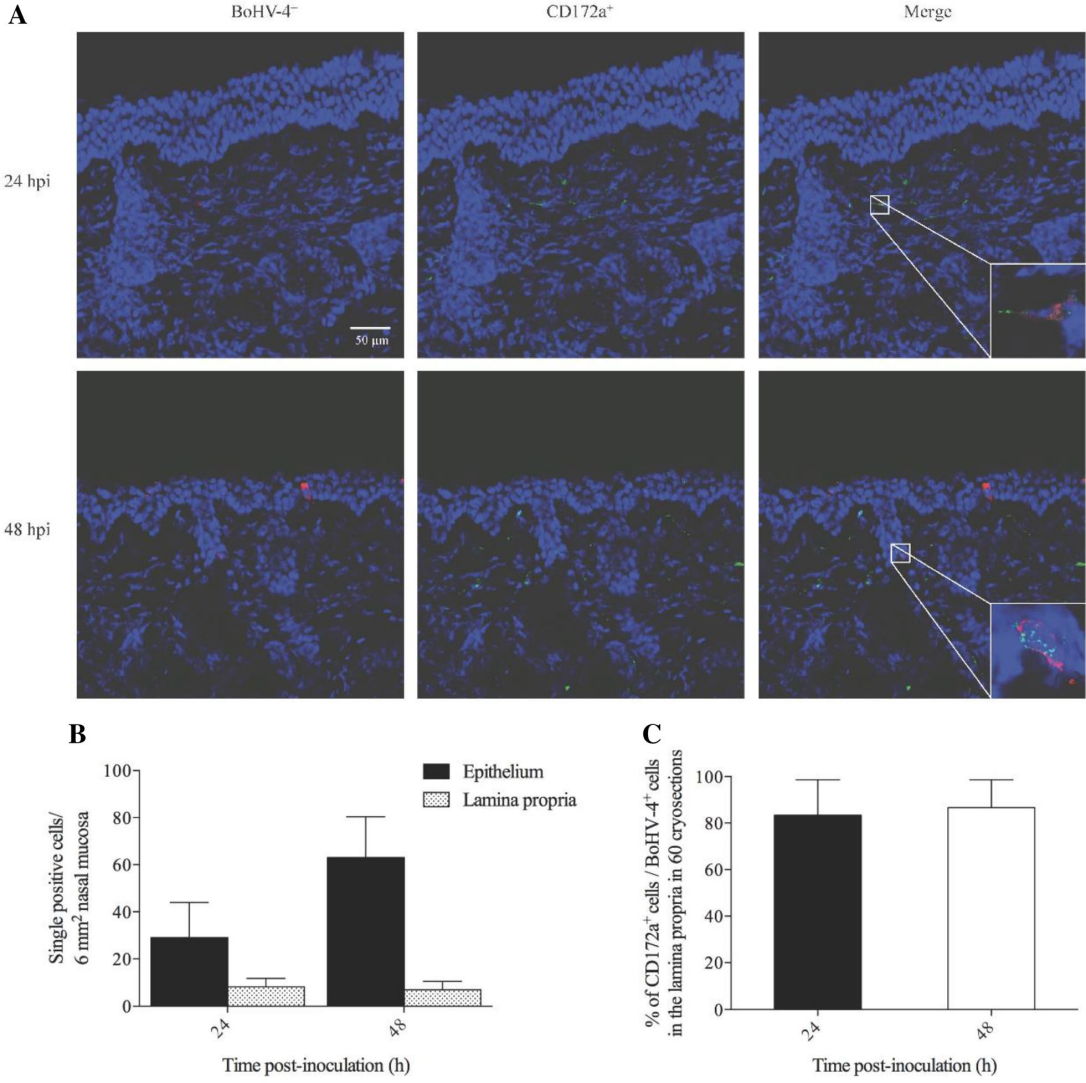
**Double immunofluorescent stainings were performed at 24** **h and 48 hpi in nasal mucosal explants.** Representative confocal immunofluorescent images show BoHV-4 (red) infected CD172a^+^ cells (green) in the lamina propria (**A**). Number of single BoHV-4 infected cells in both epithelium and lamina propria of nasal mucosa (**B**) and the percentage of CD172a^+^ cells in the lamina propria (**C**) are given per 6 mm^2^ nasal mucosa at different time points after inoculation. All data are represented as means + SD of triplicate independent experiments.

To better understand the cell tropism of BoHV-4 after inoculation, double immunofluorescent stainings were performed on the nasal mucosa explants of the respiratory tract at 24 h and 48 hpi (Figure 2A). At 24 hpi, the percentage of infected cells in the epithelium that were identified as cytokeratin^+^ was 90.2 ± 8.4%. A minority of infected cells was characterized as CD172a^+^ in the epithelium. In the lamina propria, 83.3 ± 15.2% of the infected cells were CD172a^+^ cells. Similar results were obtained at 48 hpi. The number of infected cells in the lamina propria at 48 hpi was not increased compared to the number at 24 hpi (Figures 2B and C). BoHV-4 might use CD172a^+^ monocytic cells to cross the basement membrane to reach internal target organs.

### EGTA can disrupt respiratory epithelial intercellular junctions

**Figure 3:**
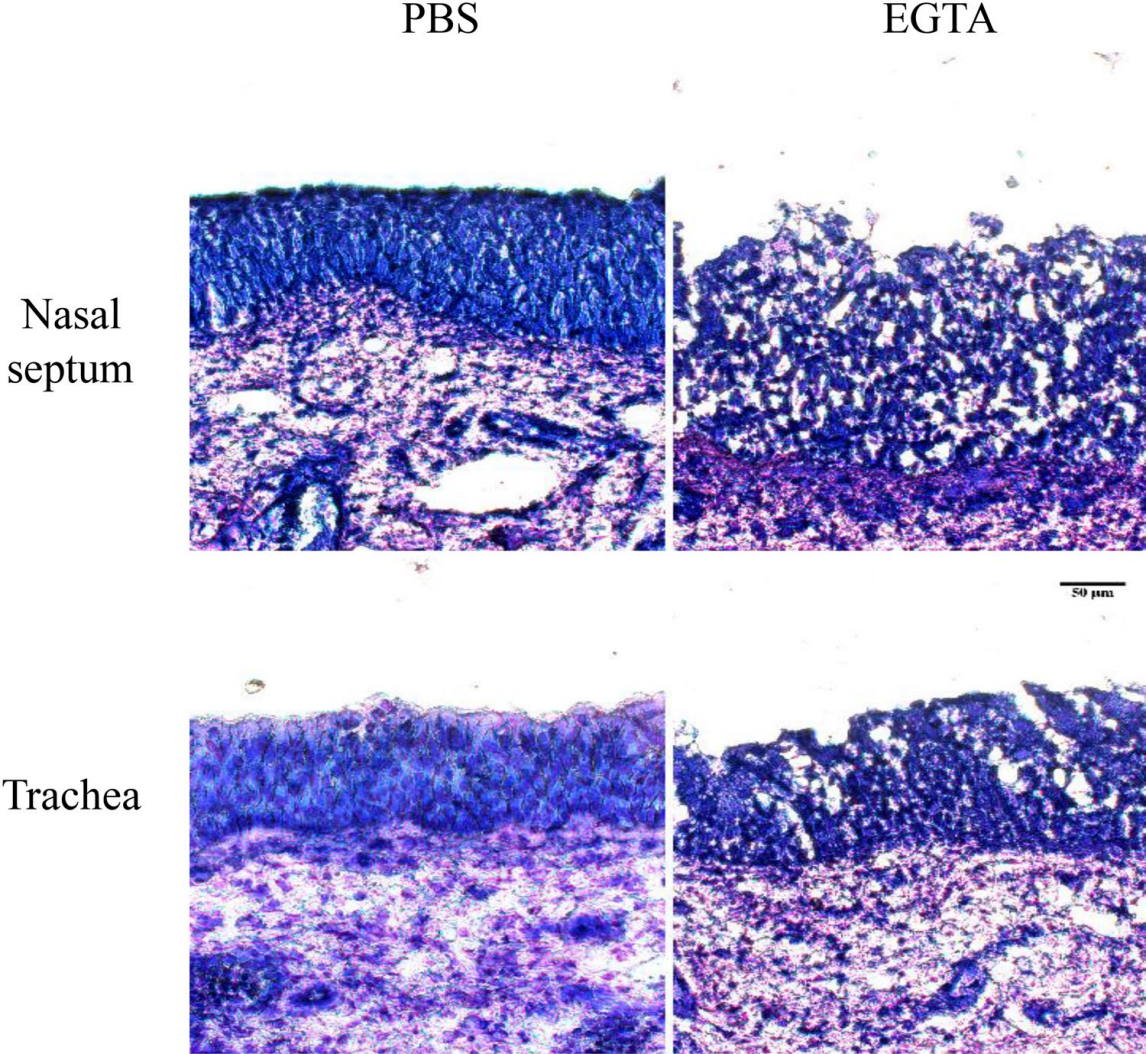
**Disruption of ICJ in respiratory mucosal explants.** Representative images of Diff-quick stained cryosections of the explants 1 h after treatment with PBS or EGTA.

The intercellular spaces in the respiratory epithelium of both nasal and tracheal mucosa explants increased after treatment with 8 mM EGTA, but not after treatment with PBS. Representative images of cryosections stained with modified Wright-Giemsa (Diff-Quick, Fisher Diagnostics, Newark, DE, USA) are shown in Figure 3. Cell viability in the respiratory mucosa explants did not significantly drop after treatment with 8 mM EGTA compared with control PBS based on TUNEL-staining (Additional file 1). EGTA treatment disrupts intercellular junctions of respiratory epithelial cells without causing significant cell death.

### Intercellular junctions protect respiratory mucosal explants to a certain extent from BoHV-4 infection

**Figure 4:**
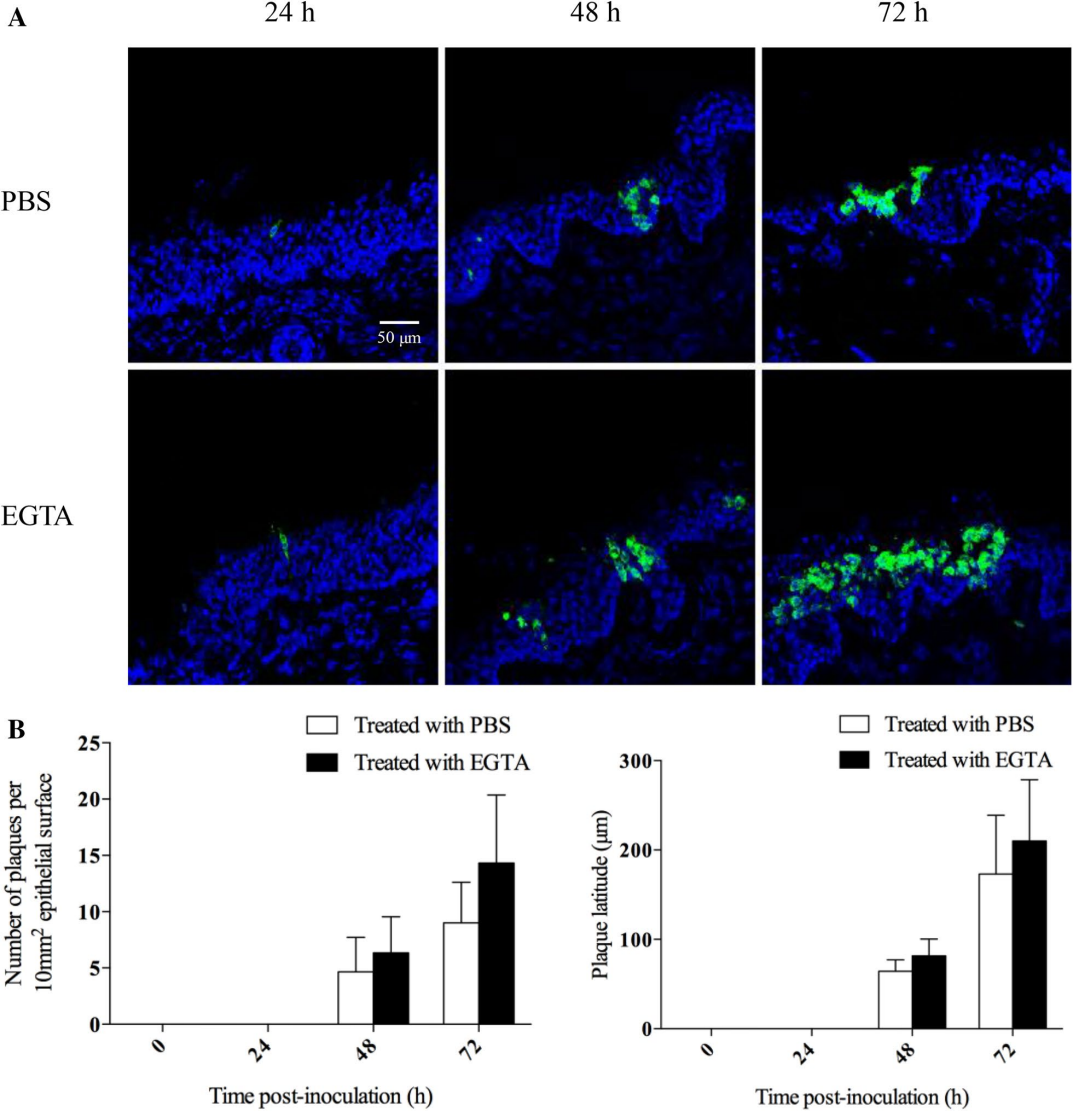
**BoHV-4 infection of respiratory mucosal explants after PBS or EGTA treatment.** Respiratory mucosal explants (nasal septum) were pre-incubated with PBS or EGTA, prior to inoculation with BoHV-4 for 1 h. Representative confocal images of BoHV-4 (green) replication at 0, 24, 48 and 72 hpi (**A**). Cell nuclei were stained with Hoechst (blue). The number of plaques and plaque latitude were calculated in nasal mucosal explants (**B**). All data are represented as means + SD of triplicate independent experiments.

**Figure 5:**
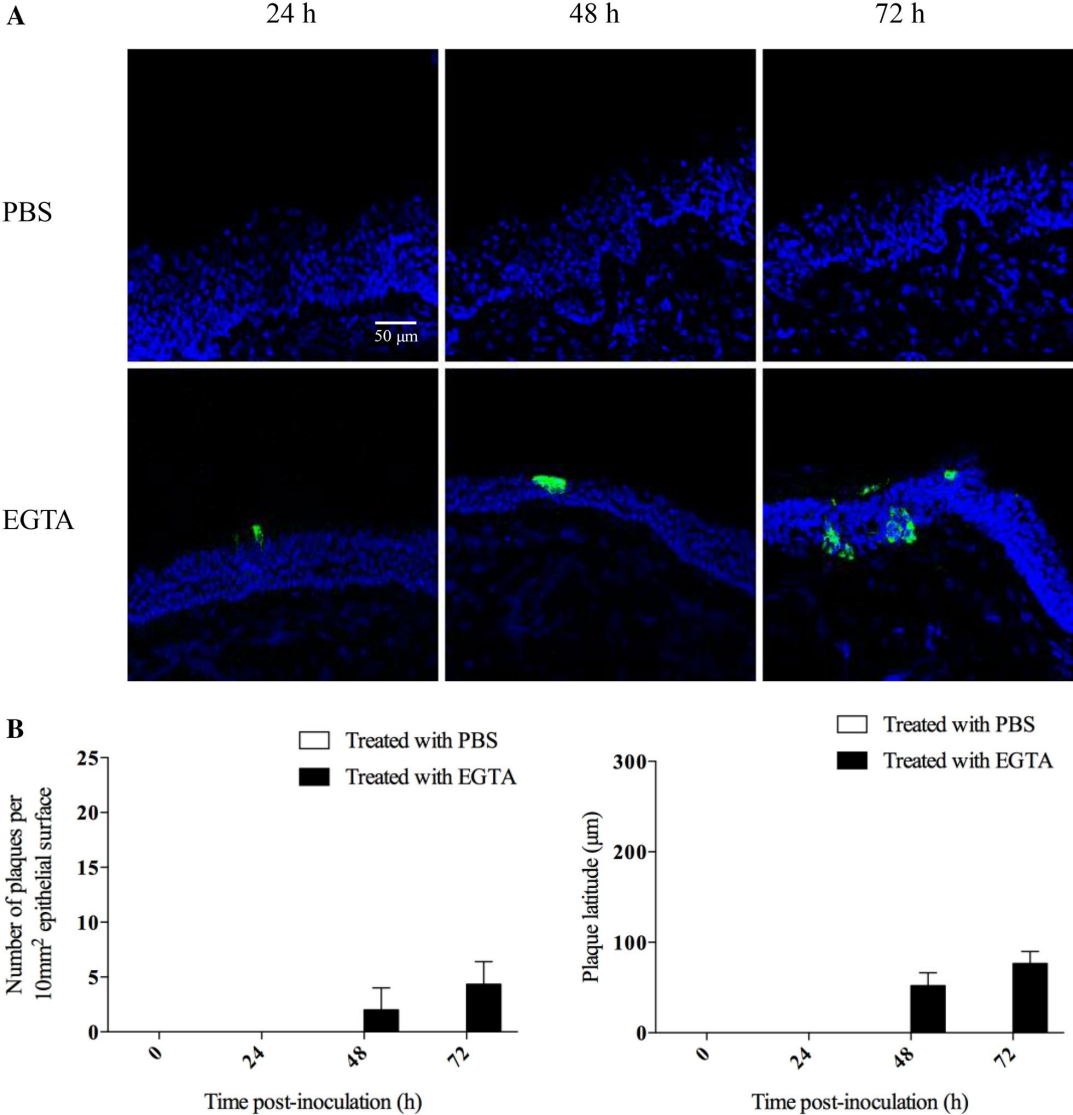
**BoHV-4 infection of respiratory mucosal explants after PBS or EGTA treatment.** Respiratory mucosal explants (trachea) were pre-incubated with PBS or EGTA, prior to inoculation with BoHV-4 for 1 h. Representative confocal images of BoHV-4 (green) replication at 0, 24, 48 and 72 hpi (**A**). Cell nuclei were stained with Hoechst (blue). The number of plaques and plaque latitude were calculated in tracheal mucosal explants (**B**). All data are represented as means + SD of triplicate independent experiments.

The number of plaques, plaque latitude and number of single cells were determined after treatment with EGTA or control PBS. Representative pictures of nasal and tracheal mucosa explants are shown in Figures 4A and 5A, respectively. As shown in Figures 4B and 5B, the number of plaques per 100 cryosections of nasal mucosa explants increased from 5 ± 3 to 6 ± 3 (*p* = 0.94) at 48 h and 9 ± 4 to 14 ± 6 (*p* = 0.15) at 72 hpi by treatment with EGTA. For tracheal mucosa explants, BoHV-4 infection was only found after treatment with EGTA. The number of plaques per 100 cryosections was 2 ± 2 at 48 h and 4 ± 2 at 72 hpi.

The plaque latitude is indicative for the lateral viral spread in the explant. For nasal mucosa explants, the average latitude increased from 64.3 ± 12.9 μm to 81.7 ± 18.8 μm (*p* = 0.99) at 48 h and 173.0 ± 65.8 μm to 210.0 ± 68.7 μm (*p* = 0.84) at 72 hpi by treatment with EGTA. For tracheal mucosal explants, the average plaque latitude in the EGTA treated explants was 52.0 ± 14.4 μm at 48 hpi and 76.7 ± 13.3 μm at 72 hpi. These results show that the integrity of ICJ protects respiratory mucosal explants to a certain extent from BoHV-4 infection.

### BoHV-4 preferentially infects basolateral surfaces of respiratory epithelial cells

**Figure 6:**
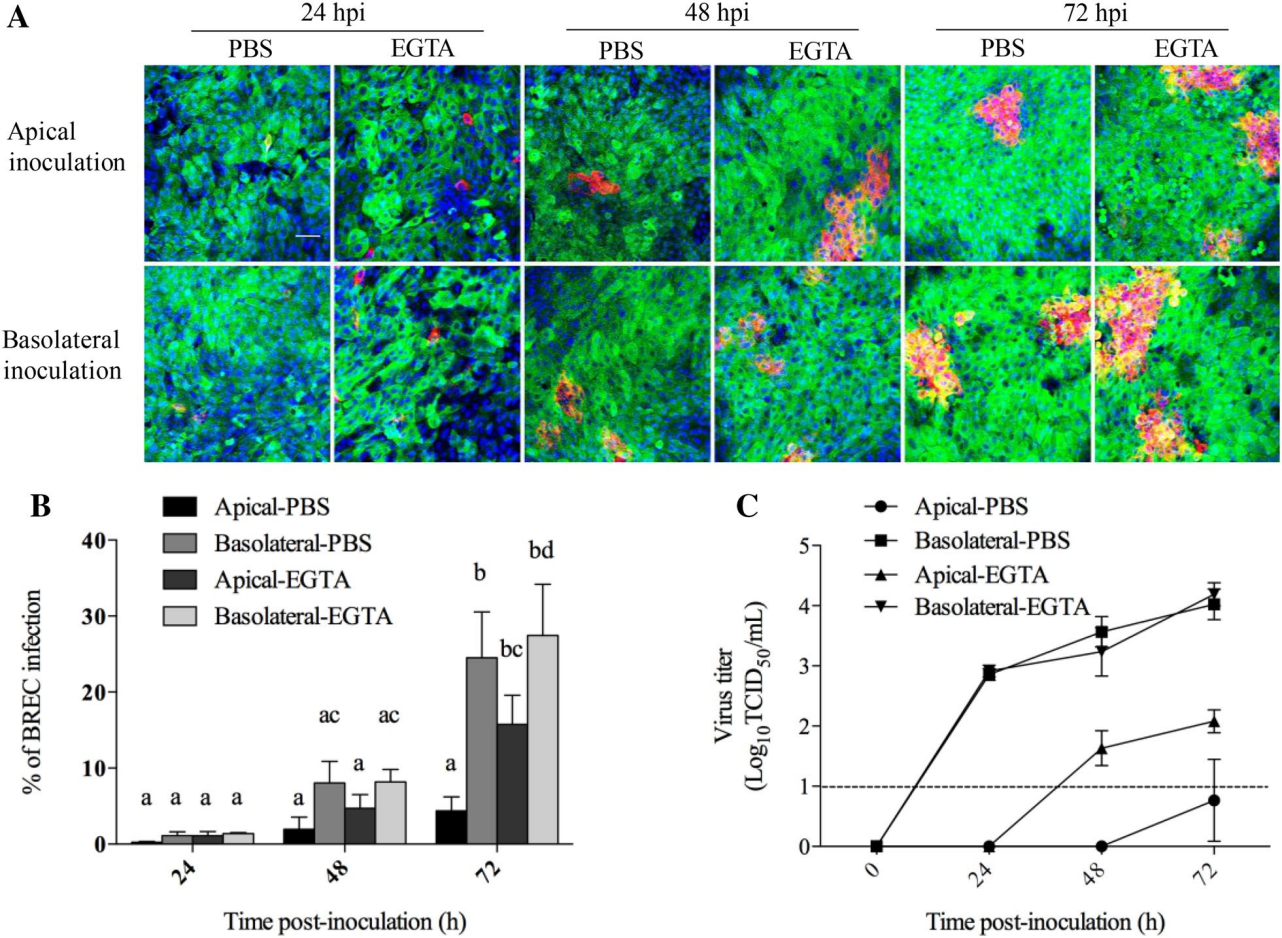
**Nasal epithelial cells were inoculated with BoHV-4 (MOI** **=** **2) at either the apical surface or basolateral surface after treatment with PBS or EGTA.** Representative confocal images of BoHV-4 (red) infection in nasal epithelial cells (green) after treatment with PBS or EGTA at 24 h, 48 h and 72 hpi; nuclei were stained with Hoechst (blue) (**A**). The scale bar represents 50 μm. The percentage of BoHV-4 infection was counted in 10 different fields of nasal epithelial cells after treatment with PBS or EGTA (**B**). In addition, the virus titers in culture medium were determined (**C**). Data are given as means + SD of triplicate independent experiments. These lower-case letters explain the p value. Significant differences (*p* < 0.05) are presented by the use of different letters.

**Figure 7:**
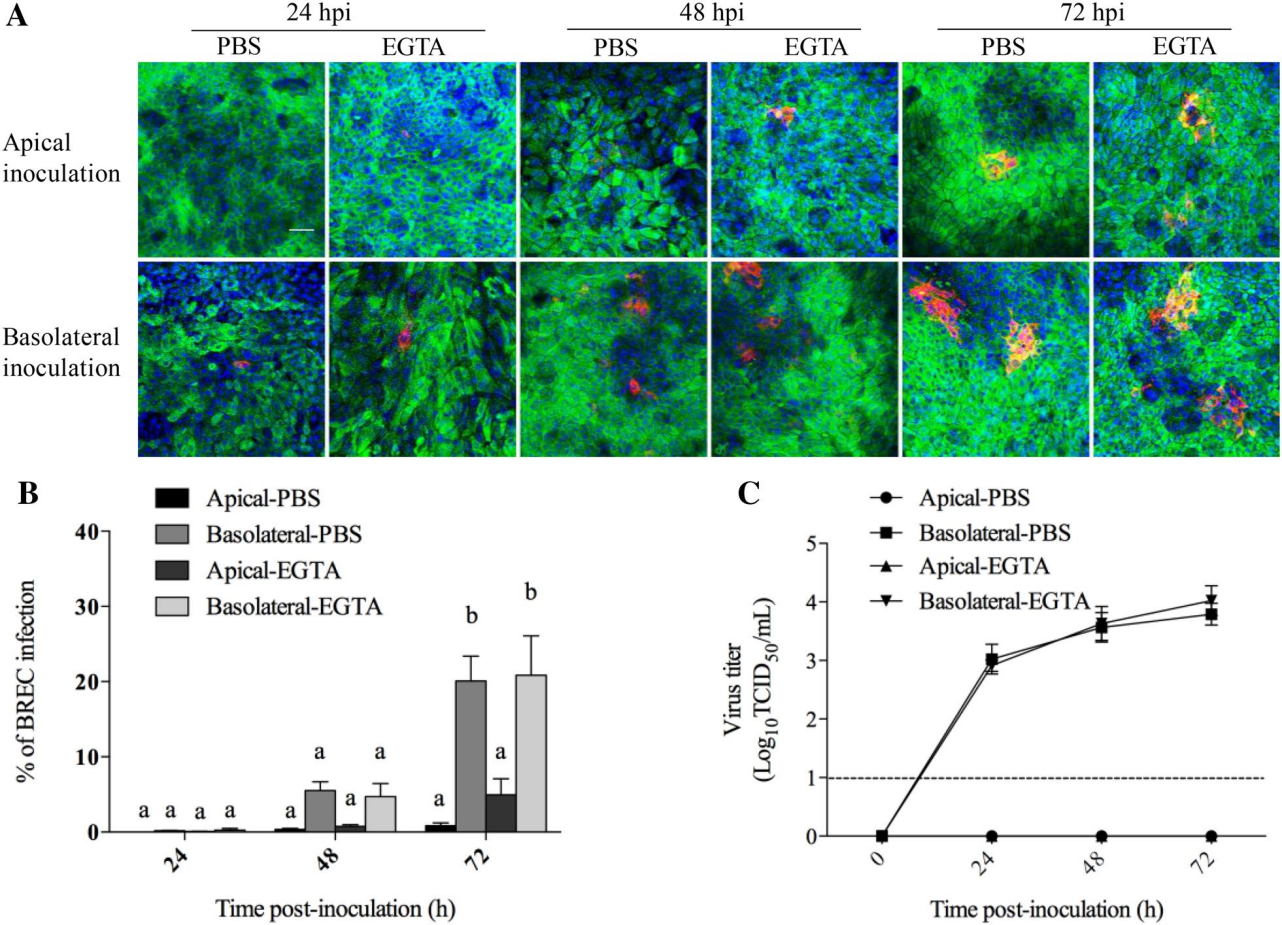
**Tracheal epithelial cells were inoculated with BoHV-4 (MOI** **=** **2) at either the apical surface or basolateral surface after treatment with PBS or EGTA.** Representative confocal images of BoHV-4 (red) infection in tracheal epithelial cells (green) after treatment with PBS or EGTA at 24 h, 48 h and 72 hpi; nuclei were stained with Hoechst (blue) (**A**). The scale bar represents 50 μm. The percentage of BoHV-4 infection was counted in 10 different fields in tracheal epithelial cells after treatment with PBS or EGTA (**B**). In addition, the virus titers in culture medium were determined (**C**). Data are given as means + SD of triplicate independent experiments. These lower-case letters explain the *p* value. Significant differences (*p* < 0.05) are presented by the use of different letters.

To investigate whether BoHV-4 preferentially infects either apical or basolateral surfaces of BREC, the percentage of infection in both nasal and tracheal epithelial cells upon apical or basolateral inoculation was evaluated at different time points (24, 48 and 72 hpi) after treatment with EGTA or PBS (mock). Representative pictures from BREC are shown in Figures 6A and 7A. For nasal epithelial cells, the average percentage of infection was 0.2 ± 0.1 at 24 h, 1.9 ± 1.6 at 48 h and 4.4 ± 1.9 at 72 hpi after mock treatment with PBS and 1.1 ± 0.6 at 24 h, 4.7 ± 1.8 at 48 h and 15.8 ± 3.8 at 72 hpi after treatment with EGTA when inoculated apically, whereas it was 1.1 ± 0.5, 8.1 ± 2.8 and 24.5 ± 6.1 after treatment with PBS and 1.4 ± 0.2, 8.2 ± 1.7 and 27.4 ± 6.8 after treatment with EGTA when inoculated basolaterally. For tracheal cells, the average percentage of infection was 0.01 ± 0.01 at 24 h, 0.3 ± 0.2 at 48 h and 0.9 ± 0.4 at 72 hpi after treatment with PBS and 0.08 ± 0.02 at 24 h, 0.7 ± 0.3 at 48 h and 4.9 ± 2.1 at 72 hpi after treatment with EGTA when inoculated apically, whereas it was 0.2 ± 0.1, 3.8 ± 0.8 and 15.1 ± 5.9 after treatment with PBS and 0.3 ± 0.2, 4.7 ± 1.8 and 20.8 ± 5.3 after treatment with EGTA when inoculated basolaterally (Figures 6B and 7B). The culture medium of inoculated BREC was collected and titrated at different time points in order to get insights in virus production and shedding. The virus titer curves are given in Figures 6C and 7C. The virus titer increased over time after basolateral inoculation of both nasal and tracheal epithelial cells. For nasal epithelial cells, the virus titer was increased after treatment with EGTA when inoculated apically, compared to treatment with PBS. These results demonstrate that basolateral inoculation of BoHV-4 results in a much higher infection in the respiratory epithelial cells than apical exposure.

### Increased epithelial cell susceptibility to BoHV-4 at the basolateral surfaces is correlated with the virus binding step

**Figure 8:**
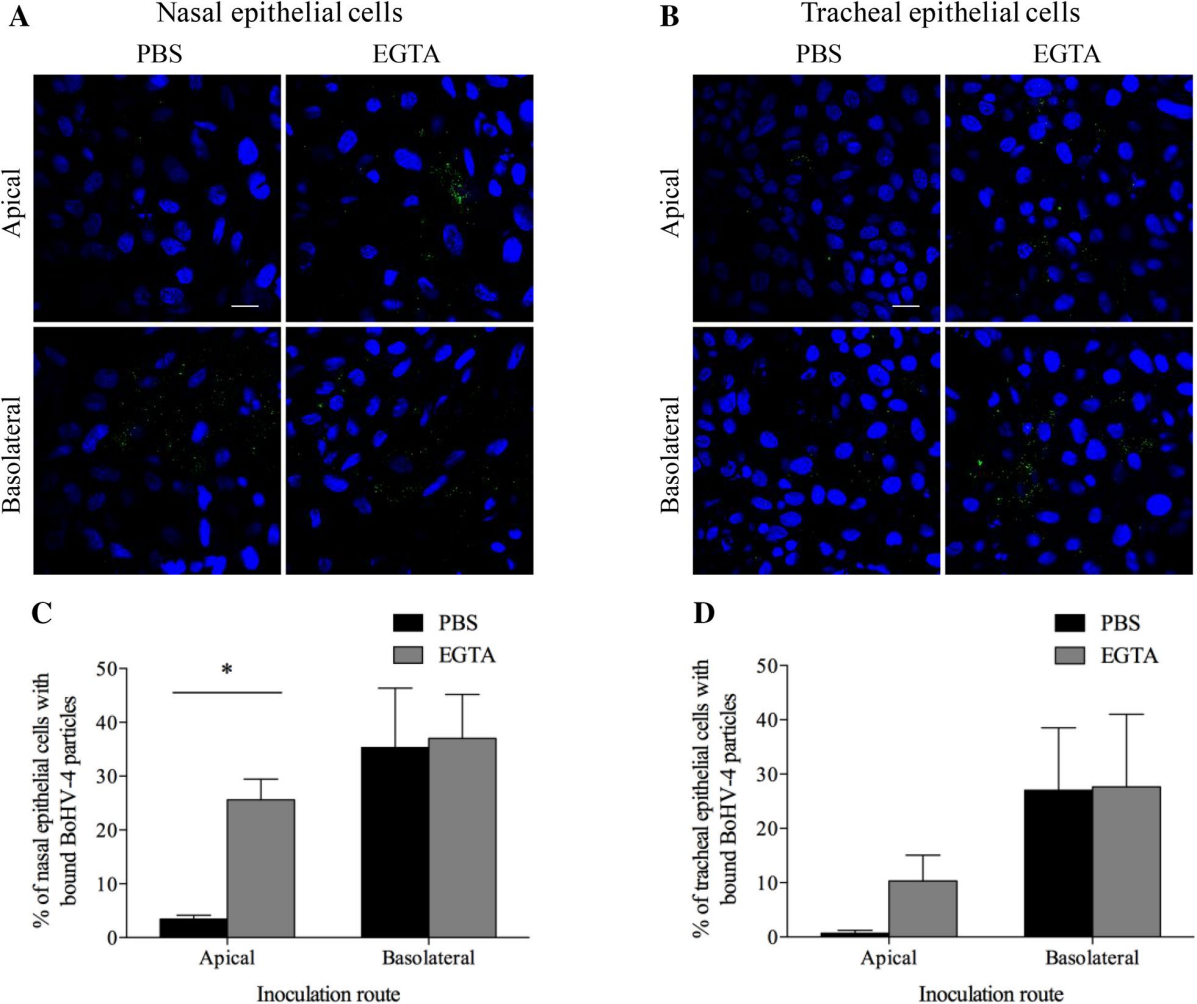
**BoHV-4 binds to nasal and tracheal epithelial cells at apical and basolateral surfaces treated with PBS or EGTA.** BREC were inoculated at 4 °C for 1 h with BoHV-4 particles (MOI = 10). Representative confocal images of nasal (**A**) and tracheal epithelial cells (**B**) with bound BoHV-4 particles (green), cell nuclei are stained in blue. The scale bar represents 20 μm. The percentage of cells with bound virus particles was calculated based on 10 random fields of 300 cells from nasal (**C**) and tracheal epithelial cells (**D**). Three independent experiments were performed and data are given as means + SD. Significant (*p* < 0.05) differences are indicated with an asterisk.

BoHV-4 binding to BREC after PBS or EGTA treatment was examined. Representative confocal images of BREC with bound BoHV-4 are shown in Figures 8A and B. The percentage of nasal epithelial cells with BoHV-4 particles after basolateral inoculation (35.3 ± 10.0) was significantly higher than after apical inoculation (3.4 ± 0.7), whereas it was 25.6 ± 3.9 upon apical inoculation and 37.0 ± 8.2 upon basolateral inoculation after treatment with EGTA. Moreover, the percentage of tracheal epithelial cells with BoHV-4 particles after the basolateral inoculation (27.0 ± 11.5) was significantly higher than after the apical inoculation (0.7 ± 0.5), whereas it was 10.3 ± 4.7 upon apical inoculation and 27.6 ± 13.4 upon basolateral inoculation after treatment with EGTA. The results are shown in Figure 8C and D. These results illustrate that BoHV-4 preferentially binds to basolateral surfaces of respiratory epithelial cells.

## Discussion

Little is known about the primary replication and dissemination of gammaherpesviruses at the host respiratory mucosal entry port. Getting a better fundamental image on how the virus behaves at its primary replication site will provide insights for prevention and treatment on a rational basis. In the present study, bovine respiratory tract mucosal explants (nasal septum and trachea) and BREC were used for studying the BoHV-4 primary infection in its host. The explant model is the perfect compromise between in vitro cell cultures and in vivo animals. It was demonstrated that BoHV-4 replicates in the nasal mucosa explants, which is in agreement with the in vivo respiratory tract symptoms associated with BoHV-4 infections. Plaques were visible in the epithelium at 48 hpi and they did not cross the basement membrane, which is corresponding with our previous findings in genital tract mucosal explants [15]. In contrast, the bovine alphaherpesvirus BoHV-1 causes an extensive epithelial plaque formation and exhibits a clear crossing of the basement membrane barrier [18]. The number and size of plaques in the nasal mucosa were slightly larger than in the vagina. This illustrates that BoHV-4 can easily use both the genital and respiratory tract for its first replication and subsequent horizontal transmission. The trachea was not found to be infected at any collection time, which may be due to the absence of apical receptors at the epithelial cells, leading to a state of resistance.

The number of single infected cells in the lamina propria was rather low and most of them were identified as CD172a^+^ cells. This indicates that BoHV-4 has a strong tropism for cells of the monocyte/macrophage lineage, which has been supported by previous studies [19–21]. Interestingly, Frederico et al. have recently used a homologous mouse model to study how the gammaherpesvirus, murine herpesvirus 4 (MuHV-4), enters the host via the upper respiratory tract [22]. They found and proposed that MuHV-4 first infects epithelial cells, then exploits myeloid cells to reach and finally infect B lymphocytes [22, 23]. BoHV-4 uses alternative splicing to express high levels of gp180 in epithelial cells but reduced levels in myeloid cells to regulate its tropism [21]. A major BoHV-4 envelope protein, gp180, is massively glycosylated [24]. Machiels et al. found that if gp180 is present, it allows efficient infection of glycosaminoglycans-positive [GAG(+)] cells like epithelial cells and if absent, it makes the virus more infectious for glycosaminoglycans-negative [GAG(-)] cells such as monocytes [25]. In our study, epithelial cells are likely the first target of BoHV-4. Next to that, monocyte/macrophages are being infected and possibly hijack the virus to cross the basement membrane, a mechanism previously described for alphaherpesvirus EHV-1 [26].

In order to examine the importance of tight junctions as a barrier for BoHV-4 infection, the respiratory mucosal explants were incubated with EGTA, a drug that disrupts the intercellular junctions. EGTA specifically induces a depletion of calcium ions from the culture medium of polarized epithelial cells which results in rapid splitting of the ICJ [27]. Recently, Van Cleemput et al. used EGTA to disrupt ICJ in equine respiratory mucosal explants and to study the impact on alphaherpesvirus EHV-1 infection [6]. The number of plaques (9 ± 4 to 14 ± 6, *p* = 0.15), plaque latitude (173.0 ± 65.8 μm to 210.0 ± 68.7 μm, *p* = 0.84) and number of single positive cells were enhanced for nasal mucosal explants by treatment with EGTA. BoHV-4 was also only able to infect the tracheal epithelium after disruption of the intercellular junctions. The infection in the trachea was significantly lower compared to the infection in nasal mucosal explants. In contrast, alphaherpesvirus EHV-1 infection was significantly lower in nasal mucosal explants than in tracheal nasal explants [6].

Since BoHV-4 infection was enhanced after disruption of the respiratory epithelium integrity with EGTA, we hypothesized that its primary binding/entry receptor is mainly located basolaterally. Therefore, BRECs were isolated and inoculated at either apical or basolateral surfaces. We found that BoHV-4 preferentially binds to and infects both nasal and tracheal epithelial cells at basolateral surfaces based on the percentage of infected cells. This is in accordance with our virus shedding when inoculated basolaterally. Virus production was increased after treatment with EGTA when inoculated apically in nasal epithelial cells. Taking together all these findings, we may conclude that BoHV-4 mainly uses a basolateral receptor. It agrees with the fact that single BoHV-4 infections are not causing severe respiratory problems, despite the power of BoHV-4 to replicate in the respiratory epithelial cells. We cannot exclude monocytic cells as primary infected cells that transfer virus to epithelial cells. These monocytic cells can also play a crucial role in transferring the virus upon reactivation. The basolateral localization of the receptor will facilitate the infection of the epithelial cells upon release of the virus from reactivated monocytic cells. One may defend the hypothesis that the epithelial cells are important both for entry and exit of the virus.

It is known that heparan sulfate (HS) serves as the initial receptor for several gammaherpesviruses. Interestingly, heparan sulfate is present on the entire surface of the membrane of undifferentiated cells, but is sequestered at the basolateral cell surface of fully differentiated cells [28, 29]. The process of adsorption of BoHV-4 is mediated by the interaction of gB with heparin-like moieties on the cell surface [30]. Furthermore, infection with the murine gammaherpesvirus MuHV-4 depends on virion binding to heparan sulfate via gp70 or gH/gL [31–33]. The human gammaherpesvirus Kaposi’s sarcoma associated herpesvirus (KSHV)-gB mediates viral binding and entry by interacting with cell surface heparan sulfate [34–36]. Previous studies examining the polarity of gammaherpesvirus infections have been conducted in continuous cell lines. To our knowledge, we were the first to use primary polarized respiratory epithelial cells to study gammaherpesvirus infections. Our results showed that the receptor is located at cellular basolateral surfaces and becomes apically accessible when intercellular junctions are impaired. The precise BREC binding/entry receptor for BoHV-4 will be identified in the near future.

In conclusion, the present study demonstrated that gammaherpesvirus BoHV-4 exhibits a specific strategy for respiratory tract invasion and subsequent spread. BoHV-4 infects epithelial cells in the nasal mucosa, rather than in the tracheal mucosa. BoHV-4 preferentially binds to and infects respiratory cells at basolateral surfaces. The virus is not able to directly cross the basement membrane; instead it hijacks CD172a^+^ monocytic cells to reach internal target organs. Taken together, our results shed new light on the primary infection of gammaherpesvirus BoHV-4.

## Additional file

**Additional file 1.** **TUNEL-staining on nasal mucosa explant after treatment with PBS or EGTA.** The TUNEL assay revealed no significant apoptotic cells (green marked with arrow bar) were caused after treatment with PBS or EGTA.
