# Supplementary figures and images for: Gammaherpesvirus BoHV-4 infects bovine respiratory epithelial cells mainly at the basolateral side
Source: Vet Res. 2019 Feb 8;50:11. doi: 10.1186/s13567-019-0629-z (PMC6368735; doi:10.1186/s13567-019-0629-z)

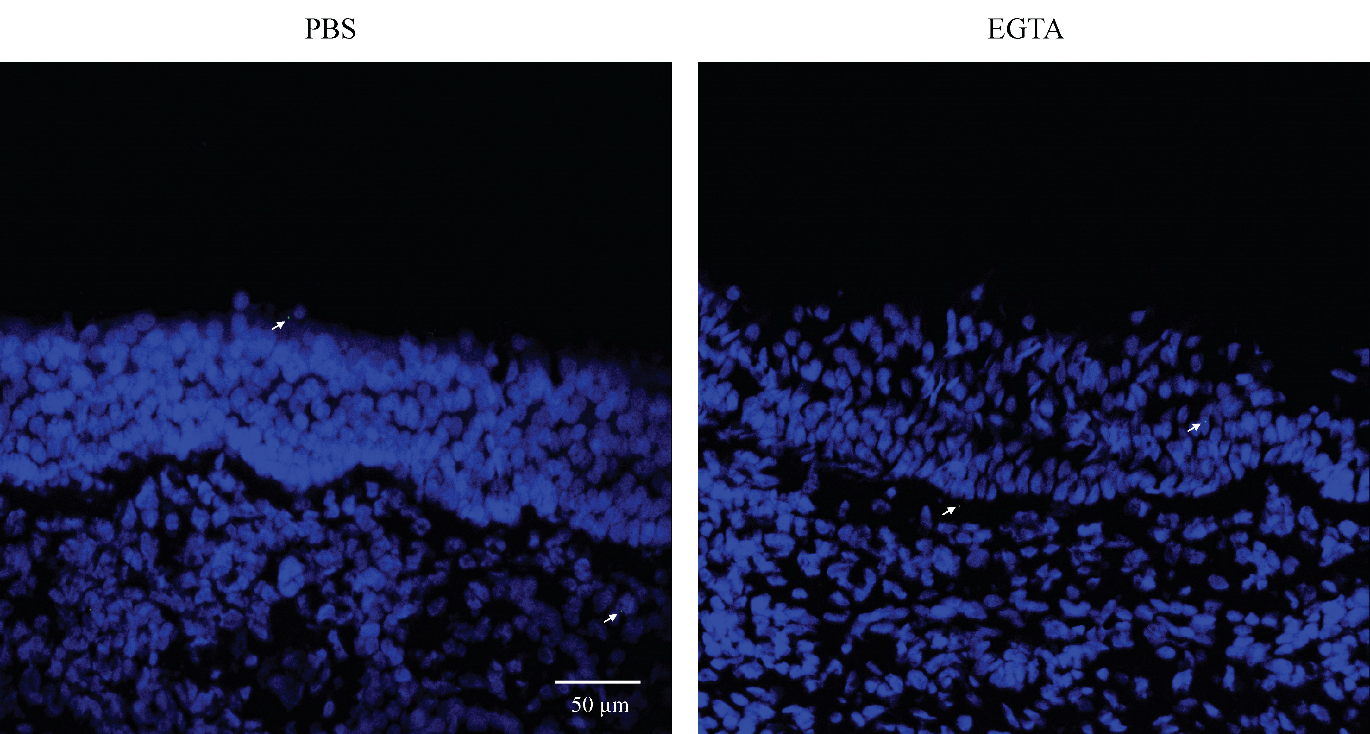

Supplement: Supplementary file 1 — Additional file 1. TUNEL-staining on nasal mucosa explant after treatment with PBS or EGTA. The TUNEL assay revealed no significant apoptotic cells (green marked with arrow bar) were caused after treatment with PBS or EGTA. [file 13567_2019_629_MOESM1_ESM.docx]
